# Exploring the Antibacterial Properties of a Newly Isolated Microviridae Phage Against Multidrug-Resistant *Escherichia coli*

**DOI:** 10.3390/pathogens15030330

**Published:** 2026-03-19

**Authors:** Yoana Kizheva, Maria Pandova, Zoltan Urshev, Yoana Gladicheva, Tsvetelina Paunova-Krasteva, Sergei Ivanov, Petya Hristova

**Affiliations:** 1 Department of General and Industrial Microbiology, Faculty of Biology, Sofia University, 1164 Sofia, Bulgaria; maria.pandova@abv.bg (M.P.); y.gladicheva@abv.bg (Y.G.); pkabad@biofac.uni-sofia.bg (P.H.); 2 RnD Department, LB Bulgaricum Plc., 1407 Sofia, Bulgaria; zoltan_urshev@yahoo.com; 3 Stephan Angeloff Institute of Microbiology, Bulgarian Academy of Science, 1000 Sofia, Bulgaria; pauny@abv.bg; 4 Research Group, Microbiological Risks in the Environment, Sofia University “St. Kliment Ohridski”, 1504 Sofia, Bulgaria; sivanov714@abv.bg

**Keywords:** phage therapy, *Microviridae* bacteriophages, *Escherichia coli*, antimicrobial resistance, MDR

## Abstract

In response to the alarming rise in antimicrobial resistance, bacteriophages have re-emerged as a promising alternative to conventional antibiotic therapy. The main objective of this paper was to characterize a newly isolated phage (vB_SEC_3) in the context of its suitability for phage therapy against MDR *E. coli*, which is considered a priority pathogen. The phage was characterized at the morphological, genomic, and biological levels relevant to phage therapy. TEM analyses revealed a non-enveloped icosahedral capsid lacking tail structure. Phylogenetic and tANI analyses placed the phage within the α3 phages (genus *Alphatrevirus*) of the less-studied family *Microviridae* and revealed <95% similarity to its closest relatives, suggesting vB_SEC_3 is a putative novel species within this genus. The genome (6085 bp, GC content 45.3%) displayed the conserved organization typical for these phages, including overlapping genes. No known genes associated with lysogeny, antibiotic resistance, or virulence were detected. Briefly, vB_SEC_3 was able to effectively lyse two MDR strains of *E. coli* (S1 and B5, EOP 0.735 and 0.961, respectively). Tolerance to a wide pH range (4–10.5) and to temperatures up to 80 °C was established. Six-month storage of the crude lysate at 4 °C resulted in a slight decrease (<0.16 log_10_ PFU/mL) in phage titer. This study provides additional insights into the biology and diversity of *Microviridae* phages and offers a basis for future investigations into their potential relevance in the context of combating MDR bacteria.

## 1. Introduction

Historical records of antibiotic discovery and their use date back to the 19th century [1]. Since then, up to the present day, the development of various antibiotic formulations and their application in the treatment of severe bacterial infections have undergone dramatic changes, ultimately resulting in the widespread emergence and development of antibiotic-resistant bacteria (ARB), primarily due to their uncontrolled and inappropriate overuse. Nowadays, antimicrobial resistance (AMR) is considered a global concern, having an impact not only on human and animal health but also on economic, social, and environmental systems [2]. Overall, water is recognized as a major pathway for the spread of AMR across humans, animals, soil, and food systems [3]. In particular, wastewater serves as a key reservoir of AMR due to the presence of antibiotic residues, resistant bacteria, and resistance genes, promoting selective pressure and horizontal gene transfer (HGT), which accelerate its dissemination [4,5].

The bacterial pathogens most frequently identified as actively involved in the transfer of AMR in wastewater include *Enterococcus faecalis*, *Staphylococcus aureus*, *Klebsiella pneumoniae*, *Acinetobacter baumannii*, *Pseudomonas aeruginosa*, *Enterobacter* spp., and *Escherichia coli* [6,7]. The latter species is a normal inhabitant of the gastrointestinal tracts (GITs) of humans and animals and is consequently present in fecal excreta. For this reason, *E. coli* has been widely used as an indicator microorganism for fecal contamination in water [8]. The species is among the leading causative agents of severe diseases and was listed by the World Health Organization (WHO) as a priority bacterial pathogen in 2024 [9]. Moreover, according to the Global Burden of Disease study, nearly 50% of AMR-attributable deaths in high-income countries are associated with *E. coli* and *S. aureus* [10]. Additionally, *E. coli* possesses multiple mechanisms that confer resistance to a wide range of antibiotics (particularly to β-lactams), resulting in multidrug resistance, and its ability to readily transfer this resistance further increases the concern [11]. Thus, in light of pessimistic projections indicating that the global mortality associated with ARB could reach ten million deaths per year by 2050 if urgent action is not taken to address the problem, new and effective treatment strategies should be developed and implemented to manage *E. coli*-associated diseases, particularly those caused by MDR strains [12]. Thus, following a decline in interest after the discovery of antibiotics, bacteriophages (phages for short) and phage therapy have regained scientific attention in recent years as promising alternatives or adjuncts to conventional antibiotic treatments.

As a target bacterial pathogen, *E. coli* is extensively investigated for the development of phage-based formulations. In brief, the majority of published studies have primarily focused on the therapeutic potential of double-stranded DNA (dsDNA) tailed bacteriophages formerly classified within the families *Siphoviridae*, *Myoviridae*, and *Podoviridae* (order *Caudovirales*), now included in the class *Caudoviricetes*, as well as on the recently established taxon within this class, the subfamily *Stephanstirmvirinae* [13,14,15,16,17,18,19]. However, *E. coli* serves as a host for a far greater diversity of phages, which, for various reasons, have remained relatively poorly studied. Such is the case, for example, with virulent single-stranded (ss) DNA (+) phages of the family *Microviridae*. Representatives of this viral family, along with crAss-like phages, are considered among the most stable colonizers of the healthy human gut phagome, as shown by longitudinal metagenomic analyses and genome-based taxonomic clustering [20]. On the other hand, summarized data indicate that in non-GIT habitats, such as wastewater, these phages occur at lower abundances than tailed dsDNA phages, typically comprising only 2% to 37% of all somatic coliphages (SC), which could explain this gap [21,22,23,24]. Members of the family *Microviridae* are classified into two subfamilies, *Gokushovirinae* and *Bullavirinae*. The latter comprises three genera that include phages infecting members of the family *Enterobacteriaceae* and *E. coli*, in particular *Alphatrevirus* (α3 phages), *Gequatrovirus* (G4 phages), and *Sinsheimervirus* (phiX174 phages) [25]. Despite their lytic nature, these phages are normal members of the human gut phagome, which likely renders them tolerated by the immune system, potentially making them less susceptible to recognition by the human immune system and subsequent excessive inflammatory responses following potential administration [26]. However, in light of the development of phage therapy against pathogenic and MDR *E. coli*, the potential of *Microviridae* phages remains relatively poorly explored, as only a limited number of studies highlighting their therapeutic potential have been reported [26,27,28].

Based on the limited data on the less-explored use of *Microviridae* phages in phage therapy against *E. coli*, the present study aimed to contribute to the existing knowledge regarding the suitability of these phages for phage therapy. The object of the study was a newly isolated phage (vB_SEC_3), found to be a member of the genus *Alphatrevirus*, family *Microviridae*. The obtained data showed that the phage possesses several desirable characteristics—lytic life cycle, clear genome (no known lysogeny, virulence, and antibiotic resistance genes were found), good tolerance to pH and temperature, good survival rate at common laboratory storage (4 °C) and, most importantly, capable of lysing MDR *E. coli* strains—which highlight its potential for application in phage therapy. Based on this, we believe that our study contributes to the global understanding of the biology and diversity of *Microviridae* phages and further advances research highlighting their potential in combating MDR bacteria.

## 2. Materials and Methods

### 2.1. Collection and Processing of the Wastewater Samples

Wastewater treatment plants (WWTPs) of three of the largest cities in Bulgaria—Sofia, Burgas, and Varna—were selected for sampling. The sampling period covered two months from February to March 2024. Influent water samples (*n* = 6, representing one monthly sample collected from each of the three sampling sites) were collected in sterile 1 L plastic containers and transported under refrigerated temperature (4 °C) to the laboratory, where they were processed immediately. The initial processing of the raw water samples was as described elsewhere [29]. After removing the large debris from the samples, they were used for the isolation of both bacteria and phages.

### 2.2. Isolation and Identification of E. coli from Wastewater

For the purposes of this study, a total of fifteen bacterial isolates, presumably belonging to the *E. coli* species, were obtained from wastewater samples (Table 1). Endo agar (EA) was used as a selective medium for the targeted isolation of *E. coli* (Merck KGaA, Darmstadt, Germany). One milliliter from each processed wastewater sample was serially 10-fold diluted in sterile saline (pH 7.0), and 100 µL aliquots from dilutions up to 10^−3^ were surface-inoculated onto EA and incubated for 24 h at 37 °C. Only single colonies exhibiting a characteristic morphology (red colonies with a permanent metallic sheen), typical of *E. coli* growth on this medium, were isolated. Pure bacterial cultures were obtained after three consecutive cultivations on non-selective medium nutrient agar (NA) (Merck KGaA, Darmstadt, Germany) and stored at −20 °C for further analyses. Subsequently, all isolates were Gram-stained, and the DNA from all isolates was extracted from overnight broth cultures in nutrient broth (NB) using a commercial Genomic DNA Purification Kit, following the manufacturer’s instructions (Thermo Fisher Scientific Inc., Waltham, MA, USA). Species identification was performed via species-specific PCR with primer pair UAL-754 (5′-AAAACGGCAAGAAAAAGCAG-3′)/UAR-900 (5′-ACGCGTGGTTACAGTCTTGCG-3′) [30]. The reaction mixture (25 µL) contained 16.5 µL sterile ultra-pure H_2_O, 6.5 µL Red Taq DNA Polymerase 2X Master Mix 2.0 mM MgCl_2_ (VWR, Darmstadt, Germany), 0.5 µL of each primer, and 1 µL DNA template. The reaction conditions were as described by Bej and colleagues [30]. The resulting amplicons were separated in 1.5% agarose gel and observed under UV light. The detection of a 147 bp product was considered reliable evidence for *E. coli* identification.

### 2.3. Phenotypic Antibiotic Susceptibility Testing of the Wastewater-Derived E. coli Isolates

The phenotypic antibiotic susceptibility (PAS) of the wastewater-derived *E. coli* isolates to seventeen antibiotics, representatives of eight antimicrobial categories (Ampicillin (AMP), 10 µg/disk; Ampicillin/Sulbactam (A/S), 10–10 µg/disk; Amoxicillin/Clavulanic acid (AMC), 20–10 µg/disk; Cefuroxime (CXM), 30 µg/disk; Imipenem (IPM), 10 µg/disk; Meropenem (MRP), 10 µg/disk; Aztreonam (AT), 30 µg/disk; Levofloxacin (LE), 5 µg/disk; Norfloxacin (NX), 10 µg/disk; Pefloxacin (PEF), 5 µg/disk; Amikacin (Am), 30 µg/disk; Gentamicin (GEN), 10 µg/disk; Eravacycline (ERV), 20 µg/disk; Tigecycline (TGC), 15 µg/disk; Fosfomycin (FO), 200 µg/disk; Nitrofurantoin (NIT), 100 µg/disk; Trimethoprim (TR), 5 µg/disk) was screened according to EUCAST recommendations (break point table version 15.0, valid from 1 January 2025) [31]. Log-phase bacterial cultures were obtained after overnight cultivation on NA at 37 °C. Bacterial suspensions were prepared in sterile saline (10^8^ CFU/mL, McFarland standard 0.5) and surface inoculated on Mueller–Hinton agar plates (MHA, Merck KGaA, Darmstadt, Germany). After placing the antibiotic-impregnated paper disks (HiMedia, Mumbai, India), the Petri dishes were cultivated at 37 °C for 16–24 h. The interpretations of the results were carried out according to EUCAST recommendations. Isolates exhibiting resistance to at least three classes of antimicrobials were considered MDR [32].

### 2.4. Bacteriophage Isolation, Purification, and Propagation

Prior to phage isolation, one wastewater sample (collected from the WWTP near Sofia in March) was passed through a 0.22 µm pore-size membrane filter (Corning Incorporated, Corning, NY 14831, USA) to obtain a bacteria-free suspension. A non-pathogenic laboratory strain of *E. coli*, NBIMCC 8432, was used as the initial host strain for bacteriophage recovery from wastewater. The bacterial strain was initially cultivated on NA for 24 h at 37 °C and subsequently used for the preparation of a bacterial suspension in sterile saline (10^8^ CFU/mL). Double agar overlay plaque assay (DAOPA) was applied for phage isolation [33]. A mixture containing equal volumes (100 µL) of bacterial suspension and filtered wastewater sample, 30 µL 1 M CaCl_2_, and 3 mL semisolid NA (0.45% agar content) was poured onto solid NA agar plates supplemented with CaCl_2_ to a final concentration of 10 mM (NA-Ca). Double agar petri dishes were cultivated for 24 h at 37 °C. A selected single plaque resulting from bacterial lysis was picked with a sterile plastic needle and transferred into 10 mL of sterile NB-Ca (10 mM CaCl_2_ content), followed by cultivation (37 °C, 24 h) with the host strain. Pure phage lysate was obtained after membrane filtration of the obtained culture through a membrane filter (pore size 0.22 µm). Phage purification was accomplished following three successive cultivations in NB-Ca with the corresponding host strain. The newly isolated phage was stored as pure lysate at 4 °C and used for further analyses.

### 2.5. Study of the Phage Host Range and Efficiency of Plating Against MDR E. coli Strains

Spot testing assay (STA) was applied to determine the host range of the newly isolated phage [34]. In total, twenty-seven bacterial strains, with representatives of eleven bacterial species (*E. coli*, *E. cloacae*, *E. asburiae*, *E. kobei*, *K. oxytoca*, *K. pneumoniae*, *A. baumannii*, *A. guillouiae*, *P. mirabilis*, *S. aureus*, and *P. aeruginosa)* were used. Fifteen of the *E. coli* strains used were isolated from wastewater in this study, and one was an ATCC strain isolated from feces (Table 1). Non-*Escherichia* species that were not obtained from publicly available repositories (ATCC, NBIMCC) used in this study were part of a laboratory collection of the Department of General and Industrial Microbiology, Sofia University “St. Kliment Ohridski”, and were previously isolated and identified (data not published). Log-phase bacterial cultures obtained after overnight cultivation on NA were used to prepare bacterial suspensions (10^8^ CFU/mL) in sterile saline. Solid NA-Ca plates were prepared, and mixtures containing 100 µL of the respective bacterial suspension, 10 mM CaCl_2_, and 3 mL semisolid NA-Ca were poured onto them. Ten µL of pure phage lysate (10^9^ PFU/mL), obtained after cultivation of the newly isolated phage with its initial host (*E. coli* 8432), was spotted onto a semisolid agar surface, and the petri dishes were cultivated at 37 °C for 24 h. The observation of the bacterial lysis area after cultivation was considered evidence for bacterial susceptibility to phage infection. The relative efficiency of plating (EOP) of the phage on susceptible *E. coli* strains was further evaluated. Briefly, double-layer agar plates were prepared as described above. The phage lysate was serially diluted tenfold in phage buffer up to 10^−7^, and 10 µL aliquots of each dilution were spotted onto the agar surface [35]. The relative EOP was calculated as the ratio of average PFU/mL obtained for the tested strain to the average PFU/mL obtained for the original host strain *E. coli* 8432 [36]. The EOP of the phage on *E. coli* 8432 was considered 1. The experiment was conducted in triplicate, and the results were expressed as mean ± SD.

### 2.6. Determination of Plaques and Virion Morphology of the Phage

Plaque morphology was examined on single plaques formed after 24 h of cultivation on *E. coli* 8432 lawn using DAOPA. The general plaque appearance (clear or turbid; presence or absence of a halo) and plaque dimensions (measured with an electronic caliper) were recorded. Measurements were conducted on at least ten individual plaques, and the final dimensions were expressed as the mean ± SD. The virion morphology was studied via transmission electron microscopy (TEM). Fifty μL of pure phage lysate (10^10^ PFU/mL) was spotted onto pre-coated Formvar grids for TEM analyses. The grids were negatively stained with 1% uranyl acetate in 70% methanol for 30 min in the dark. The observations were carried out on Hitachi HT7800, operating at 100 kV (Hitachi High-Tech Corporation, Tokyo, Japan). Phage dimensions were determined from at least ten individual measurements of single virions and were reported as mean ± SD.

### 2.7. Determination of Optimal Phage-to-Bacteria Ratio for Yielding High-Titer Lysates

The optimal phage-to-bacteria ratio, resulting in the highest phage titers (i.e., multiplicity of infection, MOI), was determined for the newly isolated phage. The phage isolate and its respective bacterial host (*E. coli* 8432) were cultivated in 10 mL NB-Ca at different ratios corresponding to MOI values of 0.01, 0.1, 1, 10, and 100. After incubation at 37 °C for 4 h, phage titers were measured, and the MOI yielding the highest titers was considered optimal for phage propagation. The experiments were carried out in triplicate, and the final titers were expressed as mean ± SD.

### 2.8. Influence of Temperature and pH on Phage Viability

Pure phage lysates with known titers were stored at 4 °C for 6 months. Phage stability under these non-specific laboratory conditions was evaluated by comparing titers at the start of the experiment with those measured after the storage period. Phage stability under various temperatures and pH was studied according to the procedures described by Park and colleagues, with slight modifications [37]. Pure phage lysates were prepared in NB-Ca, and the initial titer was counted via STA with *E. coli* 8432 as the host. Phage viability at different temperatures was evaluated by measuring titer reductions following 2 h incubation of 1 mL of pure phage lysate at 28 °C, 37 °C, 50 °C, 65 °C, 80 °C, and 95 °C, respectively. The analyses were carried out in a heating dry block under stable thermal conditions (BioSan, Riga, Latvia). The effect of pH on phage viability was evaluated by mixing 100 µL of pure phage lysate with known titer with 900 µL of phage buffer (10 mM Tris–HCl, 10 mM MgSO_4_, 68 mM NaCl, and 1 mM CaCl_2_) adjusted to pH 2.0, 4.0, 5.92, 7.5, 9.0, 10.5, and 13.0. The mixtures were incubated for 3 h, after which the number of viable phage particles was determined via STA. Reductions in phage titers were interpreted as evidence of phage susceptibility to the respective pH. The experiments were carried out in triplicate, and the final results were expressed as mean ± SD.

### 2.9. Phage Genome Sequencing and Feature Analysis

Crude phage lysate was prepared in 30 mL NB-Ca according to optimal MOI, and the titer was assessed via STA (10^9^ PFU/mL). Phage genetic material was extracted following the previously described method [29]. Sequencing was performed at Microbes NG (Birmingham, UK) using a hybrid approach that combined Oxford Nanopore Technologies (R10.4.1 flow cells) with 2 × 250 bp Illumina short reads. Raw reads were analyzed in Phage Galaxy by Kraken (Galaxy Version 1.3.1), metaviralSPAdes (Galaxy Version 4.2.0+galaxy0), and Megahit (Galaxy Version 1.2.9+galaxy2) [38,39,40,41]. The assembled genome was analyzed in CLC sequence viewer (Version 7.6) and annotated both manually and using two web-based services, Pharokka software (Galaxy Version 1.3.2) and the RAST Server: Rapid Annotations using Subsystems Technology [42,43]. The presence/absence of tRNA, virulence-associated, and antibiotic resistance genes in the phage genome was screened via tRNA prediction (tRNAscan) (Galaxy version 0.4) and ABRicate (Galaxy Version 1.0.1.), respectively [44,45].

### 2.10. Phylogenetic Analyses Based on Whole Genome Comparison

The phylogenetic relationship between the newly isolated phage and related bacteriophages was analyzed using the web-based platform VICTOR, a widely used tool for genome-based phylogeny and classification of prokaryotic viruses [46]. FASTA sequences of the complete genomes of 40 phages, members of the family *Microviridae*, were obtained from GenBank and used in the analyses. The phylogenetic tree was constructed based on pairwise comparisons of whole-genome nucleotide sequences using the Genome-BLAST Distance Phylogeny (GBDP) method with parameters recommended for prokaryotic viruses (including 100 pseudo-bootstrap replicates) [46,47]. Comparative genome alignment and the corresponding graphical representation were performed using EasyFig v.2.2.5. [48]. Sequence similarity between the genome of the newly isolated phage and its relatives was calculated using VClust v.1.2.8., a web-based tool that performs average nucleotide identity (ANI)-based genome clustering in accordance with the standards of the International Committee on Taxonomy of Viruses (ICTV) [49].

### 2.11. Statistical Analysis

The data were analyzed by Microsoft Excel v. 2508 using XLMiner Analysis ToolPak with ANOVA single factor. The number of antibiotic resistance profiles among isolates from different cities and sampling times was analyzed by Welch’s *t*-test. A *p*-value < 0.05 was considered statistically significant.

## 3. Results

### 3.1. Assessment of the Phenotypic Antibiotic Resistance Profiles of the Newly Isolated E. coli Strains

**Figure 1:**
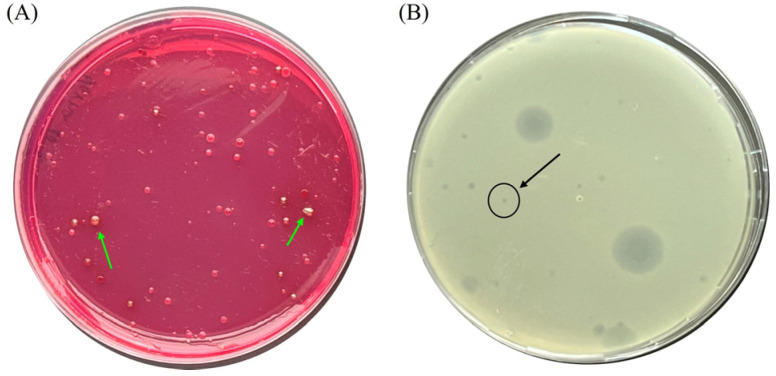
(**A**) Endo agar plate showing the bacterial growth after cultivation of the wastewater sample. The green arrows indicate the typical red colonies with a permanent metallic (fuchsin) sheen. (**B**) Plaques on double-layer NA–Ca agar plates obtained after cultivation of filtered wastewater samples with *E. coli* 8432. The black arrow indicates the plaque of origin of vB_SEC_3.

For the purposes of this study, fifteen bacterial isolates (WWTP Sofia (*n* = 6), WWTP Burgas (*n* = 6), and WWTP Varna (*n* = 3)) were obtained following cultivation of the wastewater samples on the selective EA (Table 1). Presumptive *E. coli* strains were selected based on colony morphology on EA, characterized by a red color with a permanent metallic (fuchsin) sheen, indicative of lactose fermentation (Figure 1A). All isolates were found to be Gram-negative short rods and were subsequently identified as *E. coli* based on species-specific PCR analysis, with all isolates yielding the expected 147 bp PCR product [30]. PAS testing showed that all newly isolated *E. coli* strains were susceptible to eight out of the seventeen antibiotics tested, representing five antimicrobial categories (aminoglycosides, tetracyclines, carbapenems, miscellaneous agents, and penicillin + β-lactamase inhibitors). The same strains were 100% resistant to five other substances, representing four antimicrobial categories—phosphonic acid derivative antibiotics, a unique class of antimicrobials (FO), cephalosporin antibiotics (CXM), monobactams (AT), penicillin/penicillin + β-lactamase inhibitors (AMP and AMC, respectively)—in accordance with EUCAST guidelines (Supplementary Table S1). Additionally, resistance to antibiotics in two more antimicrobial categories—quinolone/fluoroquinolone antibiotics (NX and PEF) and dihydrofolate reductase inhibitors (antifolates) (TR)—was also detected in individual isolates (B2, B6, and V7). The most antibiotic-resistant isolate was found to be V7. Based on the obtained results and in accordance with the guidelines listed in EUCAST, it can be concluded that all *E. coli* strains isolated in this study could be classified as MDR [32]. However, no statistically significant differences were observed when comparing the number of antibiotic-resistant profiles among isolates obtained from different WWTPs (cities of Sofia/Varna, Varna/Burgas, and Sofia/Burgas; *p* = 0.423, *p* = 0.788, and *p* = 0.185, respectively), nor between the two sampling time points (February and March; *p* = 0.926).

### 3.2. Bacteriophage Isolation, Host Range Determination, and EOP Evaluation

A non-pathogenic *E. coli* 8432 strain was used as the initial host for phage isolation from wastewater (Table 1). Following cultivation of the wastewater sample with the host bacterium, several plaques with distinct morphologies were observed on the surface of the double-agar plates. One small, clear plaque was chosen for phage isolation (Figure 1B). After purification via three consecutive cultivations with the host bacterium, a pure phage culture was obtained. The newly isolated phage was designated as vB_SEC_3. The host range analysis revealed that the phage can lyse only representatives of the species *E. coli*. Beyond the phage activity against its initial host (strain 8432), vB_SEC_3 was found to lyse another three strains (S1, B5, and 8739) out of the 16 tested *E. coli* strains (18.6%). No lytic activity was observed against the other tested species (Table 1). On this basis, we can conclude that vB_SEC_3 has a narrow host range. Moreover, two of the susceptible *E. coli* strains were defined as MDR (S1 and B5) based on their antibiotic resistance profiles (Supplementary Table S1). Therefore, we further evaluated the phage EOP against these two strains. The results revealed that vB_SEC_3 was highly effective against MDR strains S1 and B5, with EOP of 0.735 and 0.961, respectively. Although these differences in the original host were found to be statistically significant (*p* < 0.05), it can be concluded that the phage demonstrates high efficacy against both isolates, effectively lysing them.

### 3.3. Investigation of the Morphology of vB_SEC_3

**Figure 2:**
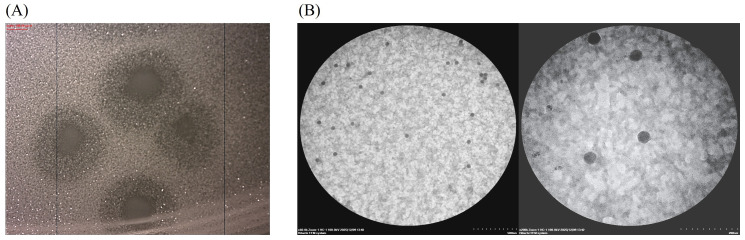
Morphological characteristics of vB_SEC_3. (**A**) Plaque morphology observed after 24 h of cultivation on NA-Ca agar with host *E. coli* 8432. Black vertical lines are generated automatically when the camera captures the image. (**B**) Virion morphology obtained by TEM. The **left TEM image** represents a general field of view (magnification ×60.0k, scale bar—500 nm). The **right TEM image** shows a clear, magnified view of individual phage particles (magnification ×200.0k, scale bar = 200 nm).

Phage’s basic characterization includes the determination of both plaque and virion morphology. The newly isolated phage formed plaques with a small clear center with a diameter (d, mm) of 1.473 ± 0.197 surrounded by a turbid zone (d = 3.215 ± 0.35 mm) on an *E. coli* 8432 lawn (Figure 2A). The virion morphology, examined by TEM, was found to be similar to that of *Microviridae* phages, displaying a small icosahedral capsid (45.16 ± 2.01 nm in diameter) with no tail structures observed (Figure 2B).

### 3.4. Determination of the Optimal MOI of the Phage

**Figure 3:**
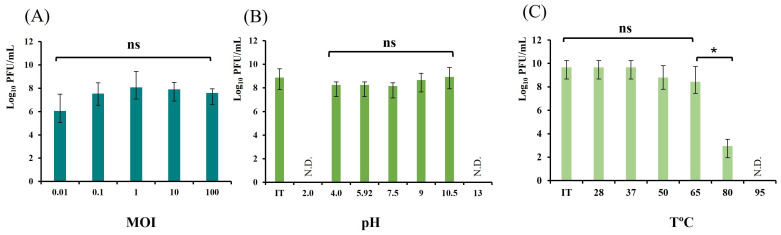
(**A**) Phage titers obtained after 4 h cultivation with host *E. coli* 8432 in NB-Ca in different phage-to-bacteria ratios (MOI). (**B**) Influence of pH on vB_SEC_3 viability. The image shows the obtained titers after 3 h incubation in pH-corrected buffers. (**C**) Thermal stability of the phage vB_SEC_3. The image shows the obtained titers after 2 h incubation at different temperatures. All values are expressed as means ± SD from three independent trials. The asterisk indicates statistical significance (*p* < 0.05), ns—statistically non-significant. IT—initial titer; N.D.—not detected.

Determination of the optimal MOI is an essential step in characterizing a newly isolated phage, as it identifies the best phage-to-bacteria ratio in the cultivation medium to achieve high-titer lysates. The comparison of the obtained average final titers with ANOVA revealed no statistical differences (*p* = 0.206). However, out of five tested ratios, we established that the highest average titer was obtained in MOI 1 (8.068 PFU/mL ± 1.36) (the ratio between phage particles and bacterial cells in the cultivation medium is equal), after 4 h cultivation with the initial host in NB-Ca (Figure 3A). Thus, this phage-to-bacterium ratio was selected as optimal and applied in subsequent phage propagation experiments where appropriate.

### 3.5. Tolerance of vB_SEC_3 to Various pH Values and Temperatures

In this study, we investigated the effect of seven pH values ranging from 2.0 to 13.0 on phage viability. Temperature tolerance was evaluated in two aspects: the effect of storage temperature (4 °C) on phage viability, and the thermal stability of virion particles across a temperature range of 28 °C to 95 °C. Generally, no viable phage particles were detected after incubation of vB_SEC_3 at extremely acidic and extremely basic conditions (pH 2.0 and 13.0). At the same time, the phage exhibited strong tolerance across a wide pH range (4.0–10.5) (Figure 3B). ANOVA analysis revealed no statistically significant differences in phage titers (*p* = 0.311) following incubation under these pH conditions, indicating that phage activity was not adversely affected. The effect of standard refrigeration conditions (4 °C) on phage viability and ability to lyse its host bacterium was studied over a six-month period by comparing phage titers measured before and after storage. The obtained results showed a small decrease in phage titer across the tested period (up to 0.16 log_10_ PFU/mL). Briefly, no statistically significant differences in phage titers compared to the initial values (9.67 ± 0.58 log_10_ PFU/mL) were observed after incubation at temperatures up to 65 °C (*p* = 0.302), indicating that this temperature range had no adverse effect on phage activity (Figure 3C). Incubation at 80 °C resulted in a significant reduction in phage titer (a decrease by 6.72 log_10_ PFU/mL) compared to the initial value. This finding was supported by ANOVA, which revealed highly significant differences in phage titers between samples incubated at 65 °C and 80 °C (*p* < 0.05). No viable phage particles were detected after incubation at 95 °C.

### 3.6. Genome Organization of vB_SEC_3

**Figure 4:**
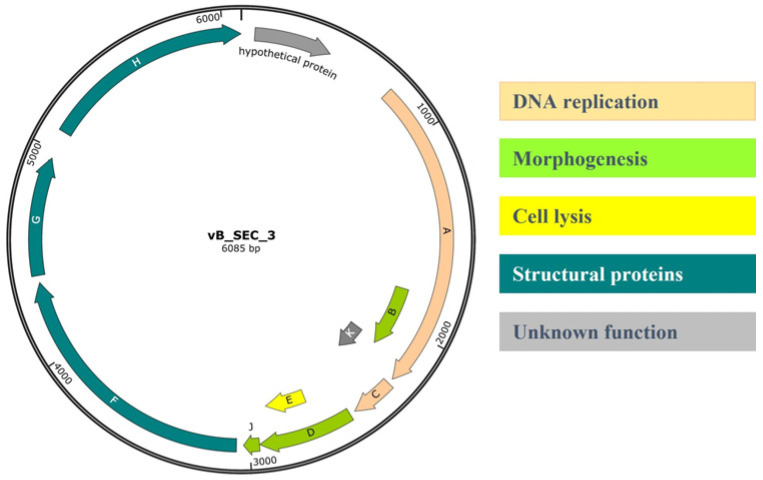
Genome map of the complete sequence of the phage vB_SEC_3. The processed sequence was deposited in GenBank with accession number PX907427. The image represents one circular fragment of ssDNA and the presence of overlapping reading frames (genes B, K, and E). Different colors indicate the annotated CDS and predicted functions of the products. The letters in the arrows indicated the predicted genes in the phage’s genome. The image was generated with SnapGene (Version 8.1.1).

**Figure 5:**
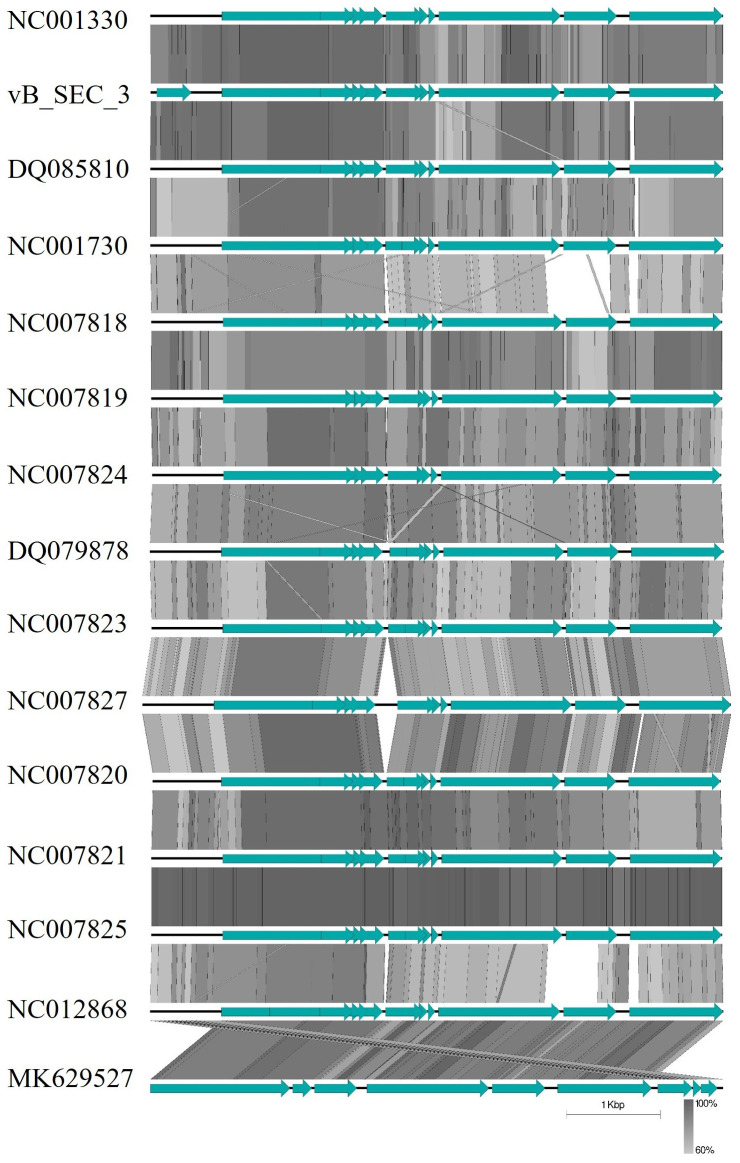
Comparative genome alignment, revealing the similarities and differences in genome organization and ORF positioning (light-green arrows) between vB_SEC_3 and other complete genomes of known α3 *Microviridae* phages deposited in GenBank. The genetic similarities are shown as percent homology (grayscale). The image was generated with the EasyFig v. 2.2.5. program.

The genetic material of the newly isolated phage was extracted, and the complete genome was sequenced, after which the obtained raw reads were processed. The total number of reads mapped with the Kraken software (Galaxy Version 1.3.1) to the *Microviridae* family was 33,742, which, after assembling, yielded a single genome with completeness of 100% with a coverage of 1663×. The annotated genome was deposited at GenBank under the accession number PX907427. The phage was found to possess one circular fragment of single-stranded DNA (+) (ssDNA) with 6085 bp length and GC content of 45.3%. The genome contained nine open reading frames (ORFs) and 11 coding sequences (CDS) and was found to possess a typical organization of known microvirus phages, with the most distinctive feature being the presence of overlapping reading frames (Figure 4). In brief, the genome of vB_SEC_3 contains three genes (B, K, and E) embedded within other CDS. These three genes were annotated as encoding protein K with unknown function, protein E, involved in cell lysis, and the highly flexible internal scaffolding protein B. The remaining seven genes were annotated as coding for products involved in virus replication (A and C), virion morphogenesis (D and J), or structural (capsid) proteins (F, G, and H). Functional annotations were derived from Kodaira and colleagues [50]. No genes associated with lysogeny, antibiotic resistance, virulence, or tRNA-related regions were identified in the genome of vB_SEC_3. Preliminary whole-genome BLASTN analysis revealed high similarity to two Enterobacteria (*Escherichia*) phages alpha3 (NC001303 and DQ085810), with nucleotide identities of 94.99% and 95.06%, respectively, and 100% query coverage. Subsequently, comparative genome alignment was performed to further analyze similarities and differences in genome organization and ORF positioning between vB_SEC_3 and other known α3 phages, revealing a high degree of genetic similarity (Figure 5).

### 3.7. Comparative Genome Analyses and Phylogenetic Relationships

**Figure 6:**
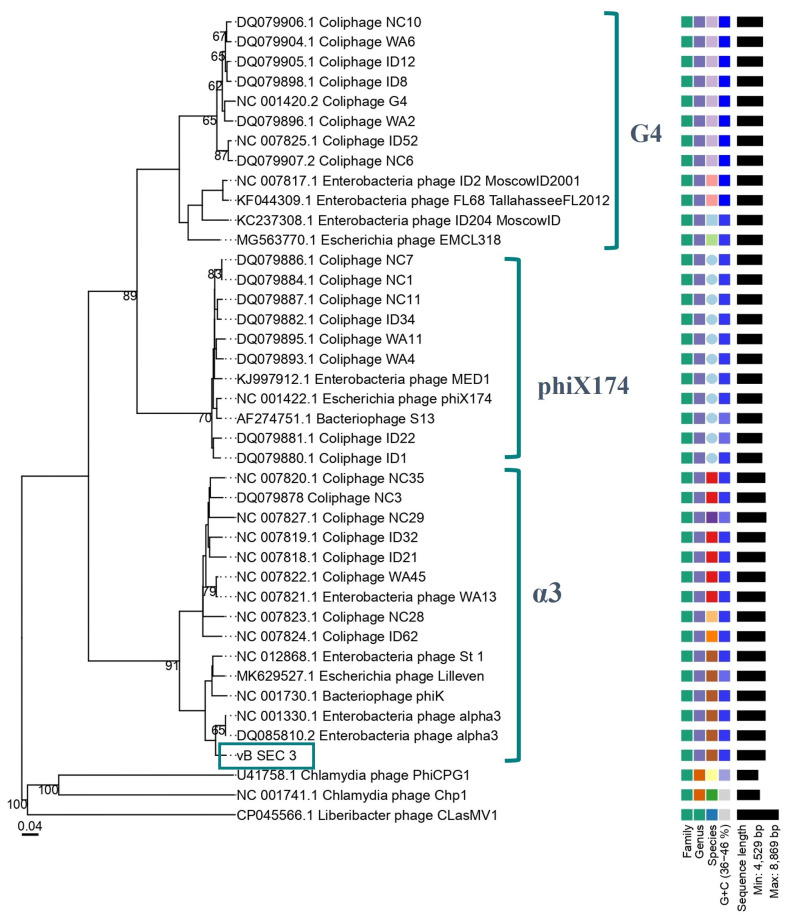
Phylogenetic tree generated based on the whole genome sequences similarity between vB_SEC_3 (dark green rectangle) and selected *Microviridae* phages, classified within α3, phiX174, and G4 groups. *Chlamydia* phage PhiCPG1, *Chlamydia* phage Chp1, and *Liberibacter* phage CLasMV1 were used as outgroups. The image was generated by the VICTOR web service (https://ggdc.dsmz.de/victor.php), accessed on 27 January 2026.

To more precisely determine the taxonomic position of the newly isolated phage, a phylogenetic tree was constructed based on pairwise comparisons of whole-genome nucleotide sequences of vB_SEC_3 and other members of the family *Microviridae* (Figure 6). Complete genome sequences of 37 bacteriophages belonging to the established groups α3, phiX174, and G4 within subfamily *Bullavirinae* were retrieved from GenBank and used for comparative analysis. The results showed that the newly isolated phage clustered together with the two phages mentioned above (NC001303 and DQ085810), revealing the phylogenetic relationship between them, thus confirming that these phages are the closest relatives of vB_SEC_3. Both phages are also known as Alphatrevirus alpha3 and are classified within the genus *Alphatrevirus*. Based on these findings, we could assume that vB_SEC_3 is a member of the group α3 phages within the family *Microviridae*, genus *Alphatrevirus*. To further validate these findings, intergenomic pairwise similarity analyses were conducted among the α3 group members (*n* = 14) included in the phylogenetic analysis. The calculated total ANI (tANI) confirmed our previous assumption, as high nucleotide identity was observed between vB_SEC_3 and the two closest relatives mentioned above (94.8% and 94.9%, respectively) (Table 2, Supplementary Figure S1). Nevertheless, despite the observed similarity percentages, which remained below 95%, vB_SEC_3 meets the ICTV criteria for genus and species demarcation (70% and 95%, respectively), suggesting it is a putative novel species within the genus *Alphatrevirus*.

## 4. Discussion

The rapid global increase in AMR, alongside the declining pipeline of effective antibiotics, has prompted renewed interest in alternative treatments, notably phage therapy [51]. Supporting global efforts to combat AMR and advancing phage therapy—both worldwide and locally—requires comprehensive, coordinated research, beginning with the exploration of diverse bacteriophages and the evaluation of their therapeutic potential against targeted bacterial pathogens. While this rediscovered approach has gained increased visibility and experienced rapid growth in Western regions, phage research in countries such as Bulgaria has accelerated over the past several years, building on earlier, largely isolated investigations [52]. In this regard, the present study describes the characterization of a newly isolated bacteriophage (vB_SEC_3) and presents data on its capacity to target and lyse *E. coli*, with a particular focus on MDR strains.

Influent wastewater (WWTP, Sofia, Bulgaria) was used as the source for phage isolation, consistent with other studies reporting the isolation and characterization of phages targeting enterobacteria, particularly *E. coli* [19,28,51,53,54]. A non-pathogenic *E. coli* strain (8432) was used as the initial host for phage isolation to maximize the chances of phage discovery, avoiding the naturally occurring resistance often observed in wild *E. coli* strains from the same habitat, as previously suggested [53]. The host range of the phage was examined across a wide range of test bacteria, most of which were representatives of the family *Enterobacteriaceae.* For the purposes of this study, alongside phage isolation, a total of fifteen *E. coli* isolates were obtained from influent wastewater and included in the host range analyses. The tested PAS of all newly isolated *E. coli* strains revealed that all of them exhibit MDR, primarily showing resistance to β-lactam antibiotics (CXM, AT, AMP, and AMC) along with FO. According to analyses from the Antimicrobial Testing Leadership and Surveillance (ATLAS) project, resistance to cephalosporins is central to classifying these strains as critical pathogens [55]. Additionally, the observed resistance to FO—an antibiotic previously recommended for treating urinary tract infections caused by extended-spectrum β-lactamase (ESBL- producing bacteria, and generally exhibiting low resistance rates—raises concerns about the efficacy of currently available effective antibiotics against MDR *E. coli* strains [56,57]. In this regard, we established that although vB_SEC_3 exhibited a narrow host range (targeting only *E. coli* strains), it was able to target two MDR *E. coli* isolates (S1 and B5), showing EOP > 0.5 (0.735 and 0.961, respectively) relative to the initial non-pathogenic strain (8432). According to the widely accepted EOP scale, in which phage infectivity is classified as high (EOP ≥ 0.5), moderate (0.2–0.5), low (0.001–0.2), and inefficient (≤0.001), the newly isolated phage exhibited a high EOP, highlighting its potential against MDR *E. coli* strains [58].

The genotypic and morphological characterization of vB_SEC_3 revealed that it possessed features typical of the known α3 phages, members of the genus *Alphatrevirus*, subfamily *Bullavirinae*, family *Microviridae.* These are small, non-enveloped, ssDNA (+) phages that exhibit a highly conserved genome organization and exclusively target members of the *Enterobacteriaceae* family [59,60]. Our observations established that vB_SEC_3 possessed a small icosahedral capsid with no visible tail structures, typical of *Microviridae* phages, although its dimensions (d = 45.16 ± 2.01 nm) were larger than previously reported [24,61]. Indeed, our morphological analyses were performed using TEM, following the standard pipeline for phage characterization, whereas most studies examining capsid structure, assembly, and mature virions of *Microviridae* phages employ primarily cryo-electron microscopy (cryo-EM). Notably, virion diameters measured via cryo-EM are typically reported in angstroms, which could partly explain this discrepancy [27,28,62]. Sequencing analyses revealed that the genome organization of vB_SEC_3 was highly similar to that previously reported for the *Microviridae* phages [50]. The taxonomic position of the newly isolated phage was confirmed through phylogenetic and comparative genome alignment analyses, which identified its two closest relatives—NC001303 and DQ085810—both members of the genus *Alphatrevirus*, family *Microviridae.* Moreover, based on the calculated tANI and the criteria established by the ICTV, vB_SEC_3 has the potential to be considered a putative novel species within this genus. Interestingly, the phylogenetic analysis based on the whole genome sequence similarity positioned the new vB_SEC_3 phage in one of two distinctive subclusters of α3 phages (one represented by the reference genome, acc. No. NC001303, the other by coliphage NC35, acc. No. NC007820) (Figure 6). Similarly, two distinct groups of α3 phage genome sequences, one of which included vB_SEC_3, were observed when comparing them by tANI calculation (Table 2, Supplementary Figure S1). These two analyses suggest possible intrageneric diversification within α3-like phages, an observation that merits further attention.

Additionally, the genome features of vB_SEC_3 were studied in the context of its suitability for application in phage therapy; no known genes for lysogeny, tRNA, virulence, or antibiotic resistance were established. The gene encoding phage lysis protein (gene E) was also established in the genome of vB_SEC_3. Interestingly, ssDNA phages mediate cell lysis in a manner fundamentally different from the endolysin–holin system used by dsDNA phages, although this process remains poorly explored [63]. Gene E encodes a membrane protein that inhibits MraY, an enzyme essential for murein synthesis, functioning similarly to penicillin antibiotics, which are known to inhibit cell wall synthesis [64]. Given its universal presence and essential role in bacterial survival, MraY is a promising target for antibacterial drug development [65]. With the rise of resistance to β-lactam antibiotics (also observed in our analyses) through the development of ESBL-producing bacteria, it is intriguing to consider whether MraY inhibitors, such as those encoded by the *Microviridae* phages, could offer therapeutic benefits against these resistant strains. Further research is warranted, as these phages show promising potential against ESBL-producing bacteria.

Nevertheless, despite their potential against MDR bacteria, these phages have been studied mostly in terms of their distribution, role in host-phage interactions in the human and animal gut, and genetic diversity, rather than their therapeutic applications, with few limited exceptions [26,27,28,66,67,68,69]. This discrepancy may arise because standard DNA isolation and sequencing protocols often fail to detect small ssDNA genomes, as successful isolation using conventional methods requires the phage DNA to be in its double-stranded form, which represents a transient stage in the replication strategy of ssDNA phages (known as rolling circle) [61]. In contrast, our investigation focused primarily on general phage characteristics most frequently studied for phages intended for phage therapy. The newly isolated phage was assessed for long-term viability under non-specific laboratory conditions (4 °C), as well as for thermal inactivation and pH stability. Despite its distinct biology, the newly isolated phage exhibited propagation patterns similar to those of the more extensively studied dsDNA-tailed phages, including its stability across a wide pH range of 4.0–10.5 and thermal inactivation following incubation at temperatures of 65–80 °C [29,58]. We established that only a slight decrease in phage titer occurred after storage of the crude phage lysate for 6 months at 4 °C, and that the phage produced a high number of progeny within 4 h at an MOI of 1. Such rapid production of progeny, facilitated by the small genome size and the phage’s employed replication strategy, can be considered desirable for phages intended for phage therapy. On the other hand, such phages are more prone to mutations, and although they are considered obligately lytic, in the context of phage therapy, genetic variability and potential adaptation dynamics should be monitored [61].

## 5. Conclusions

In the rapidly evolving field of strategies aimed at combating antibiotic resistance among pathogenic bacteria, particularly through bacteriophage-based approaches, this study provides new data highlighting the potential of the newly isolated phage vB_SEC_3 against MDR *E. coli* strains. As a representative of the relatively understudied family *Microviridae* in the context of phage therapy, the obtained results support further investigation of *Microviridae* phages as potential antibacterial agents. Moreover, the bacterial pathogen addressed in this study is among those of greatest interest for the development of phage-based therapies, as highlighted in the WHO report on antibacterial agents in clinical and preclinical development. A reference to this document shows that Annex 13, focusing on phage-based products and phage-derived enzymes, indicates that three out of ten reviewed products are designed to target *E. coli* [70]. In Bulgaria, previous investigations into the lytic potential of *E. coli* phages have been limited and sporadic, conducted decades ago, and the present study provides new data to advance this field [71]. However, further in-depth research is essential, particularly with regard to the phage’s efficacy against clinical MDR *E. coli* strains, the potential for resistance development, its pharmacodynamic and pharmacokinetic properties, the evaluation of endotoxins and enterotoxins in phage lysates, and cytotoxicity, before it can be considered for clinical application.

## Figures and Tables

**Table 1:** Host range of the newly isolated bacteriophage vB_SEC_3.

№	Test Bacteria	Source, Location and Period of Isolation of the Test Bacteria	vB_SEC_3 Activity
***Escherichia coli***			
1.	S1	wastewater, Sofia, February	+
2.	S2	wastewater, Sofia, February	-
3.	S3	wastewater, Sofia, February	-
4.	S5	wastewater, Sofia, March	-
5.	S6	wastewater, Sofia, March	-
6.	S7	wastewater, Sofia, March	-
7	B1	wastewater, Burgas, February	-
8.	B2	wastewater, Burgas, February	-
9.	B3	wastewater, Burgas, February	-
10.	B5	wastewater, Burgas, February	+
11.	B6	wastewater, Burgas, February	-
12.	B8	wastewater, Burgas, March	-
13.	V1	wastewater, Varna, February	-
14.	V7	wastewater, Varna, March	-
15.	V8	wastewater, Varna, March	-
16.	8739	feces, ATCC *	+
**17.**	**8432**	NBIMCC **	+
**non-*Escherichia* strains**			
18.	*Enterobacter cloacae* M1	street food, LC ***	-
19.	*Enterobacter asburiae* M3	street food, LC	-
20.	*Enterobacter kobei* M4	street food, LC	-
21.	*Klebsiella oxytoca* M5	street food, LC	-
22.	*K. pneumoniae* M7	street food, LC	-
23.	*A. baumannii* M9	street food, LC	-
24.	*Acinetobacter guillouiae* M2	street food, LC	-
25.	*Proteus mirabilis* M11	street food, LC	-
26.	*S. aureus* 6538	lesion, ATCC	-
27.	*P. aeruginosa* 9027	OEI ****, ATCC	-

* ATCC—American Type Culture Collection, ** NBIMCC—National Bank for Industrial Microorganisms and Cell Cultures; *** LC—laboratory collection of the Department of General and Industrial Microbiology, Faculty of Biology, Sofia University “St. Kliment Ohridski”, Bulgaria; bold font—the initial host for phage isolation from wastewater, **** OEI—Outer ear infection, type strain.

**Table 2:** Total ANI between α3 phages investigated in this study.

	NC007827	DQ079878	NC012868	MK629527	DQ085810	NC001730	NC001330	NC007819	NC007818	NC007821	NC007822	NC007823	vB_SEC_3	NC007824	NC007820
NC007827	100	90.2	69.9	69.2	72.4	69.2	72.1	90.1	89.6	89.5	89.2	86.7	70.0	87.6	89.5
DQ079878	90.2	100	70.5	69.6	72.0	70.0	71.9	92.1	91.2	94.4	94.2	89.3	71.9	90.2	93.2
NC012868	69.9	70.5	100	94.9	89.9	93.5	89.9	72.0	69.3	74.1	74.1	69.8	89.4	69.8	71.9
MK629527	69.2	69.6	94.9	100	89.9	92.9	89.8	69.9	67.4	71.9	70.9	69.6	89.2	69.6	73.3
DQ085810	72.4	72.0	89.9	89.9	100	87.6	99.9	73.6	70.4	75.2	73.8	71.5	94.9	70.7	72.7
NC001730	69.2	70.0	93.5	92.9	87.6	100	87.6	70.9	70.8	71.6	72.0	68.5	87.3	69.8	69.5
NC001330	72.1	71.9	89.9	89.8	99.9	87.6	100	73.4	70.2	75.1	73.8	71.6	94.8	70.6	72.7
NC007819	90.1	92.1	72.0	69.9	73.6	70.9	73.4	100	92.9	93.8	93.5	89.5	70.7	90.4	92.2
NC007818	89.6	91.2	69.3	67.4	70.4	70.8	70.2	92.9	100	91.6	91.4	89.1	70.5	89.5	90.3
NC007821	89.5	94.4	74.1	71.9	75.2	71.6	75.1	93.8	91.6	100	99.3	91.6	72.4	91.7	92.5
NC007822	89.2	94.2	74.1	70.9	73.8	72.0	73.8	93.5	91.4	99.3	100	91.5	70.7	91.9	92.2
NC007823	86.7	89.3	69.8	69.6	71.5	68.5	71.6	89.5	89.1	91.6	91.5	100	70.2	89.9	88.9
vB_SEC_3	70.0	71.9	89.4	89.2	94.9	87.3	94.8	70.7	70.5	72.4	70.7	70.2	100	72.7	71.8
NC007824	87.6	90.2	69.8	69.6	70.7	69.8	70.6	90.4	89.5	91.7	91.9	89.9	72.7	100	89.8
NC007820	89.5	93.2	71.9	73.3	72.7	69.5	72.7	92.2	90.3	92.5	92.2	88.9	71.8	89.8	100

The highlighted cells indicate the percentage identity between the closely related phage genomes.

## Data Availability

The data presented in this research are available in the manuscript. The complete genome of the phage vB_SEC_3, along with the corresponding annotation, is freely available from GenBank under accession number PX907427 (https://www.ncbi.nlm.nih.gov/nuccore/3151285872, accessed on 1 February 2026).

## Supplementary Materials

The following supporting information can be downloaded at: https://www.mdpi.com/article/10.3390/pathogens15030330/s1, Table S1. Phenotypic antibiotic resistance profiles of *E. coli* strains isolated from wastewater in this study; Figure S1. Heatmap showing the calculated tANI between vB_SEC_3 and the complete genome sequences of selected α3 *Microviridae* phages deposited in GenBank. The nucleotide similarity is expressed in percentage and illustrated from light-blue color (60%) to dark-blue color (100%). The image was generated by VClust web service (https://afproject.org/vclust/), accessed on 17 January 2026.

## References

[1] Durand G.A., Raoult D., Dubourg G. (2019). Antibiotic discovery: History, methods and perspectives. Int. J. Antimicrob..

[2] Sambaza S.S., Naicker N. (2023). Contribution of wastewater to antimicrobial resistance: A review article. J. Glob. Antimicrob. Resist..

[3] Prestinaci F., Pezzotti P., Pantosti A. (2015). Antimicrobial resistance: A global multifaceted phenomenon. Pathog. Glob. Health.

[4] Barancheshme F., Munir M. (2018). Strategies to combat antibiotic resistance in the wastewater treatment plants. Front. Microbiol..

[5] Amato M., Dasí D., González A., Ferrús M.A., Castillo M.Á. (2021). Occurrence of antibiotic-resistant bacteria and resistance genes in agricultural irrigation waters from Valencia city (Spain). Agric. Water Manag..

[6] Petrovich M.L., Zilberman A., Kaplan A., Eliraz G.R., Wang Y., Langenfeld K. (2020). Microbial and viral communities and their antibiotic resistance genes throughout a hospital wastewater treatment system. Front. Microbiol..

[7] Marano R.B., Gupta C.L., Cozer T., Jurkevitch E., Cytryn E. (2021). Hidden resistome: Enrichment reveals the presence of clinically relevant antibiotic resistance determinants in treated wastewater-irrigated soils. Environ. Sci. Technol..

[8] Abdelgalel R.R., Ibrahem R.A., Mohamed D.S., Ahmed A.B.F. (2025). Multidrug-resistant *Escherichia coli* in wastewater sources: A comparative study and identification of resistance hotspots. BMC Microbiol..

[9] (2024). Bacterial Priority Pathogens List, 2024: Bacterial Pathogens of Public Health Importance to Guide Research, Development and Strategies to Prevent and Control Antimicrobial Resistance.

[10] Murray C.J., Ikuta K.S., Sharara F., Swetschinski L., Aguilar G.R., Gray A., Han C., Bisignano C., Rao P., Wool E. (2022). Global burden of bacterial antimicrobial resistance in 2019: A systematic analysis. Lancet.

[11] Johnson T.J., Logue C.M., Johnson J.R., Kuskowski M.A., Sherwood J.S., Barnes H.J. (2012). Associations between multidrug resistance, plasmid content, and virulence potential among extraintestinal pathogenic and commensal *Escherichia coli* from humans and poultry. Foodborne Pathog. Dis..

[12] de Kraker M.E.A., Stewardson A.J., Harbarth S. (2016). Will 10 Million People Die a Year due to Antimicrobial Resistance by 2050?. PLoS Med..

[13] Korf I.H.E., Meier-Kolthoff J.P., Adriaenssens E.M., Kropinski A.M., Nimtz M., Rohde M., van Raaij M.J., Wittmann J. (2019). Still Something to Discover: Novel Insights into *Escherichia coli* Phage Diversity and Taxonomy. Viruses.

[14] Jurczak-Kurek A., Gąsior T., Nejman-Faleńczyk B., Bloch S., Dydecka A., Topka G., Miernikiewicz P., Węgrzyn A., Węgrzyn G. (2016). Biodiversity of bacteriophages: Morphological and biological properties of a large group of phages isolated from urban sewage. Sci. Rep..

[15] Nikulin N., Nikulina A., Zimin A., Aminov R. (2023). Phages for treatment of *Escherichia coli* infections. Prog. Mol. Biol. Transl. Sci..

[16] Markusková B., Elnwrani S., Andrezál M., Sedláčková T., Szemes T., Slobodníková L., Kajsik M., Drahovská H. (2024). Characterization of bacteriophages infecting multidrug-resistant uropathogenic *Escherichia coli* strains. Arch. Virol..

[17] International Committee on Taxonomy of Viruses (ICTV). https://ictv.global/taxonomy/taxondetails?taxnode_id=202407120&taxon_name=Caudoviricetes.

[18] Turner D., Shkoporov A.N., Lood C., Millard A.D., Dutilh B.E., Alfenas-Zerbini P., van Zyl L.J., Aziz R.K., Oksanen H.M., Poranen M.M. (2023). Abolishment of morphology-based taxa and change to binomial species names: 2022 taxonomy update of the ICTV bacterial viruses subcommittee. Arch. Virol..

[19] Kaneko T., Uchiyama J., Osaka T., Tsuneda S. (2025). Novel *Escherichia coli* phages representing a distinct genus within the subfamily *Stephanstirmvirinae*: Genome and host range characteristics. Arch. Virol..

[20] Shkoporov A.N., Clooney A.G., Sutton T.D.S., Ryan F.J., Daly K.M., Nolan J.A., McDonnell S.A., Khokhlova E.V., Draper L.A., Forde A. (2019). The Human Gut Virome Is Highly Diverse, Stable, and Individual Specific. Cell Host Microbe.

[21] Muniesa M., Lucena F., Jofre J. (1999). Study of the potential relationship between the morphology of infectious somatic coliphages and their persistence in the environment. J. Appl. Microbiol..

[22] Cantalupo P.G., Calgua B., Zhao G., Hundesa A., Wier A.D., Katz J.P., Grabe M., Hendrix R.W., Girones R., Wang D. (2011). Raw sewage harbors diverse viral populations. mBio.

[23] Aw T.G., Howe A., Rose J.B. (2014). Metagenomic approaches for direct and cell culture evaluation of the virological quality of wastewater. J. Virol. Methods.

[24] Bichet M.C., Gardette M., Das Neves B., Challant J., Erbs A., Roman V., Robin M., La Carbona S., Gantzer C., Boudaud N. (2024). A new understanding of somatic coliphages belonging to the *Microviridae* family in urban wastewater. Water Res..

[25] International Committee on Taxonomy of Viruses (ICTV). https://ictv.global/taxonomy/taxondetails?taxnode_id=202403855&taxon_name=Microviridae.

[26] Romeyer Dherbey J., Bertels F. (2024). The untapped potential of phage model systems as therapeutic agents. Virus Evol..

[27] Ghosh S., Persad E., Shiue T.Y., Lam C., Islam A., Mascibroda L.G., Sherman M.B., Smith T., Cheeptham N. (2018). Explorative Study on Isolation and Characterization of a *Microviridae* G4 Bacteriophage, EMCL318, against Multi-Drug-resistant *Escherichia coli* 15-318. Antibiotics.

[28] Hu W., Liu Z., Wei Y., Bian Q., Lan W., Fan C., Song J., Sun Q., Zhang X., Liu Y. (2025). Structural basis for *Salmonella* infection by two *Microviridae* phages. Commun. Biol..

[29] Kizheva Y., Dimova T., Pandova M., Gladicheva Y., Petrova R., Paunova-Krasteva T., Urshev Z., Ivanov S., Hristova P. (2025). Characterization of wastewater-derived bacteriophages infecting *Enterococcus faecalis* in Bulgaria: Insights into the novel phage vB_SEF_8. Front. Microbiol..

[30] Bej A.K., DiCesare J.L., Haff L., Atlas R.M. (1991). Detection of *Escherichia coli* and *Shigella* spp. in water by using the polymerase chain reaction and gene probes for uid. Appl. Environ. Microbiol..

[31] The European Committee on Antimicrobial Susceptibility Testing (2025). Breakpoint Tables for Interpretation of MICs and Zone Diameters. Version 15.0. https://www.eucast.org.

[32] Magiorakos A.P., Srinivasan A., Carey R.B., Carmeli Y., Falagas M.E., Giske C.G., Harbarth S., Hindler J.F., Kahlmeter G., Olsson-Liljequist B.J. (2012). Multidrug-resistant, extensively drug-resistant and pandrug-resistant bacteria: An international expert proposal for interim standard definitions for acquired resistance. Clin. Microbiol. Infect..

[33] Kropinski A.M., Mazzocco A., Waddell T.E., Lingohr E., Johnson R.P., Clokie M.R.J., Kropinski A.M. (2009). Enumeration of bacteriophages by double agar overlay plaque assay. Bacteriophages: Methods and Protocols, Vol. 1: Isolation, Characterization, and Interactions.

[34] Kutter E., Clokie M.R.J., Kropinski A.M. (2009). Phage host range and efficiency of plating. Bacteriophages: Methods and Protocols, Vol. 1: Isolation, Characterization, and Interactions.

[35] Msimbira L.A., Jaiswal S.K., Dakora F.D. (2016). Identification and characterization of phages parasitic on bradyrhizobia nodulating groundnut (*Arachis hypogaea* L.) in South Africa. Appl. Soil Ecol..

[36] Abedon S.T., Katsaounis T.I., Clokie M., Kropinski A., Lavigne R. (2018). Basic Phage Mathematics. Bacteriophages, Methods in Molecular Biology, Volume III.

[37] Park S.Y., Kwon H., Kim S.G., Park S.C., Kim J.H., Seo S. (2023). Characterization of two lytic bacteriophages, infecting *Streptococcus bovis/equinus* complex (SBSEC) from Korean ruminant. Sci. Rep..

[38] Ramsey J., Rasche H., Maughmer C., Criscione A., Mijalis E., Liu M., Hu J.C., Young R., Gill J.J. (2020). Galaxy and Apollo as a biologist-friendly interface for high-quality cooperative phage genome annotation. PLoS Comput. Biol..

[39] Wood D.E., Salzberg S.L. (2014). Kraken: Ultrafast metagenomic sequence classification using exact alignments. Genome Biol..

[40] Antipov D., Korobeynikov A., McLean J.S., Pevzner P.A. (2015). hybridSPAdes: An algorithm for hybrid assembly of short and long reads. Bioinformatics.

[41] Li D., Liu C.-M., Luo R., Sadakane K., Lam T.-W. (2015). MEGAHIT: An ultra-fast single-node solution for large and complex metagenomics assembly via succinct de Bruijn graph. Bioinformatics.

[42] Bouras G., Nepal R., Houtak G., Psaltis A.J., Wormald P.-J., Vreugde S. (2022). Pharokka: A fast scalable bacteriophage annotation tool. Bioinformatics.

[43] Aziz R.K., Bartels D., Best A.A., DeJongh M., Disz T., Edwards R.A., Formsma K., Gerdes S., Glass E., Kubal M. (2008). The RAST Server: Rapid Annotations using Subsystems Technology. BMC Genom..

[44] Lowe T.M., Eddy S.R. (1997). tRNAscan-SE: A Program for Improved Detection of Transfer RNA Genes in Genomic Sequence. Nucleic Acids Res..

[45] Seemann T. ABRicate: Mass Screening of Contigs for Antibiotic Resistance Genes. https://github.com/tseemann/abricate.

[46] Meier-Kolthoff J.P., Göker M. (2017). VICTOR: Genome-based phylogeny and classification of prokaryotic viruses. Bioinformatics.

[47] Meier-Kolthoff J.P., Auch A.F., Klenk H.-P., Göker M. (2013). Genome sequence-based species delimitation with confidence intervals and improved distance functions. BMC Bioinform..

[48] Sullivan M.J., Petty N.K., Beatson S.A. (2011). Easyfig: A genome comparison visualizer. Bioinformatics.

[49] Zielezinski A., Gudyś A., Barylski J., Siminski K., Rozwalak P., Dutilh B.E., Deorowicz S. (2025). Ultrafast and accurate sequence alignment and clustering of viral genomes. Nat. Methods.

[50] Kodaira K., Nakano K., Okada S., Taketo A. (1992). Nucleotide sequence of the genome of the bacteriophage alpha 3: Interrelationship of the genome structure and the gene products with those of the phages, phi X174, G4 and phi K. Biochim. Biophys. Acta.

[51] Nale J.Y., Chan B., Nnadi N.E., Cheng J.K.J., Matts S., Nezam-Abadi N., Turkington C.J.R., Charreton L.M., Bola H., Nazir R. (2023). Novel *Escherichia coli*-Infecting Bacteriophages Isolated from Uganda That Target Human Clinical Isolates. Phage.

[52] Kalvatchev N., Khanbabapour T., Sakkeer A., Tsekov I., Delchev Y., Strateva T. (2025). The Forgotten History of Bacteriophages in Bulgaria: An Overview and Molecular Perspective on Their Role in Addressing Antibiotic Resistance and Therapy. Viruses.

[53] Olsen N.S., Forero-Junco L., Kot W., Hansen L.H. (2020). Exploring the Remarkable Diversity of Culturable *Escherichia coli* Phages in the Danish Wastewater Environment. Viruses.

[54] Ismael N.M., Azzam M., Abdelmoteleb M., El-Shibiny A. (2024). Phage vB_Ec_ZCEC14 to treat antibiotic-resistant *Escherichia coli* isolated from urinary tract infections. Virol. J..

[55] Al-Mustapha A.I., Muasa B., Adetunji V., Awoyale O.D., Adesiyan I.M., Adesiji Y.O., Elelu N., Odetokun I.A., Ogundijo O.A., Yahaya R. (2025). Global distribution and incidence of multidrug resistant and ESBL producing *Escherichia coli*: An observational study of the ATLAS dataset. Clin. Epidemiol. Glob. Health.

[56] Gupta V., Rani H., Singla N., Kaistha N., Chander J. (2013). Determination of Extended-Spectrum β-Lactamases and AmpC Production in Uropathogenic Isolates of *Escherichia coli* and Susceptibility to Fosfomycin. J. Lab. Physicians.

[57] Ríos E., Del Carmen López Diaz M., Culebras E., Rodríguez-Avial I., Rodríguez-Avial C. (2022). Resistance to fosfomycin is increasing and is significantly associated with extended-spectrum β-lactamase-production in urinary isolates of *Escherichia coli*. Med. Microbiol. Immunol..

[58] Pazhouhnia S., Bouzari M., Arbabzadeh-Zavareh F. (2022). Isolation, characterization and complete genome analysis of a novel bacteriophage vB_EfaS-SRH2 against *Enterococcus faecalis* isolated from periodontitis patients. Sci. Rep..

[59] International Committee on Taxonomy of Viruses (ICTV). https://ictv.global/report_9th/ssDNA/Microviridae.

[60] McKenna R., Xia D., Willingmann P., Ilag L.L., Krishnaswamy S., Rossmann M.G., Olson N.H., Baker T.S., Incardona N.L. (1992). Atomic structure of single-stranded DNA bacteriophage phi X174 and its functional implications. Nature.

[61] Kirchberger P.C., Ochman H. (2023). Microviruses: A World Beyond phiX174. Annu. Rev. Virol..

[62] Mietzsch M., Kailasan S., Bennett A., Chipman P., Fane B., Huiskonen J.T., Clarke I.N., McKenna R. (2024). The Structure of Spiroplasma Virus 4: Exploring the Capsid Diversity of the Microviridae. Viruses.

[63] Chamakura K.R., Young R. (2020). Single-gene lysis in the metagenomic era. Curr. Opin. Microbiol..

[64] Zeng X., Lin J. (2013). Beta-lactamase induction and cell wall metabolism in Gram-negative bacteria. Front. Microbiol..

[65] Yamamoto K., Sato T., Hao A., Asao K., Kaguchi R., Kusaka S., Ruddarraju R.R., Kazamori D., Seo K., Takahashi S. (2024). Development of a natural product optimization strategy for inhibitors against MraY, a promising antibacterial target. Nat. Commun..

[66] Creasy A., Rosario K., Leigh B.A., Dishaw L.J., Breitbart M. (2018). Unprecedented Diversity of ssDNA Phages from the Family Microviridae Detected within the Gut of a Protochordate Model Organism (*Ciona robusta*). Viruses.

[67] Yang P., Zhang H., Yin L., Chen J., Chen Y., Yang H., Liu Q., Zhang W. (2025). Genetic diversity of *Microviridae* phages in the human respiratory tract. Front. Cell. Infect. Microbiol..

[68] Wu Y., Wu Z., Guo L., Shao J., Xiao H., Yang M., Deng C., Zhang Y., Zhang Z., Zhao Y. (2024). Diversity and distribution of a prevalent Microviridae group across the global oceans. Commun. Biol..

[69] Federici S., Kviatcovsky D., Valdés-Mas R., Elinav E. (2023). Microbiome-phage interactions in inflammatory bowel disease. Clin. Microbiol. Infect..

[70] (2025). Analysis of Antibacterial Agents in Clinical and Preclinical Development: Overview and Analysis 2025.

[71] Savov D., Karaivanov L., Todorova L. (1977). Lysogeny in *Escherichia coli* isolated from birds and the spectrum of the lytic action of isolated phage. Vet. Med. Nauki.

