# Supplementary material for: Exploring the Antibacterial Properties of a Newly Isolated Microviridae Phage Against Multidrug-Resistant Escherichia coli
Source: Pathogens. 2026 Mar 19;15(3):330. doi: 10.3390/pathogens15030330 (PMC13029530; doi:10.3390/pathogens15030330)
Supplement: Supplementary file 1 [file pathogens-15-00330-s001.zip › Supplementary Table S1.pdf]

Supplementary Table S1 Phenotypic antibiotic resistance profiles of *E. coli* strains isolated from wastewater in this study.

***E. coli* strains**	**EUCAST interpretation**																
	**Am**	**LE**	**NX**	**ERV**	**TGC**	**GEN**	**PEF**	**MRP**	**FO**	**TR**	**NIT**	**IPM**	**CXM**	**AT**	**AMP**	**A/S**	**AMC**
S1	S	S	S	S	S	S	S	S	R	S	S	S	R	R	R	S	R
S2	S	S	S	S	S	S	S	S	R	S	S	S	R	R	R	S	R
S3	S	S	S	S	S	S	S	S	R	S	S	S	R	R	R	S	R
S5	S	S	S	S	S	S	S	S	R	S	S	S	R	R	R	S	R
S6	S	S	S	S	S	S	S	S	R	S	S	S	R	R	R	S	R
S7	S	S	S	S	S	S	S	S	R	S	S	S	R	R	R	S	R
B1	S	S	S	S	S	S	S	S	R	S	S	S	R	R	R	S	R
B2	S	SI	R	S	S	S	R	S	R	S	S	S	R	R	R	S	R
B3	S	S	S	S	S	S	S	S	R	S	S	S	R	R	R	S	R
B5	S	S	S	S	S	S	S	S	R	S	S	S	R	R	R	S	R
B6	S	S	R	S	S	S	R	S	R	S	S	S	R	R	R	S	R
B8	S	S	S	S	S	S	S	S	R	S	S	S	R	R	R	S	R
V1	S	S	S	S	S	S	S	S	R	S	S	S	R	R	R	S	R
V7	S	SI	R	S	S	S	R	S	R	R	S	S	R	R	R	S	R
V8	S	S	S	S	S	S	S	S	R	S	S	S	R	R	R	S	R

R – resistant; S – susceptible; SI – susceptible, increased exposure
