# Supplementary figures and images for: Exploring the Antibacterial Properties of a Newly Isolated Microviridae Phage Against Multidrug-Resistant Escherichia coli
Source: Pathogens. 2026 Mar 19;15(3):330. doi: 10.3390/pathogens15030330 (PMC13029530; doi:10.3390/pathogens15030330)

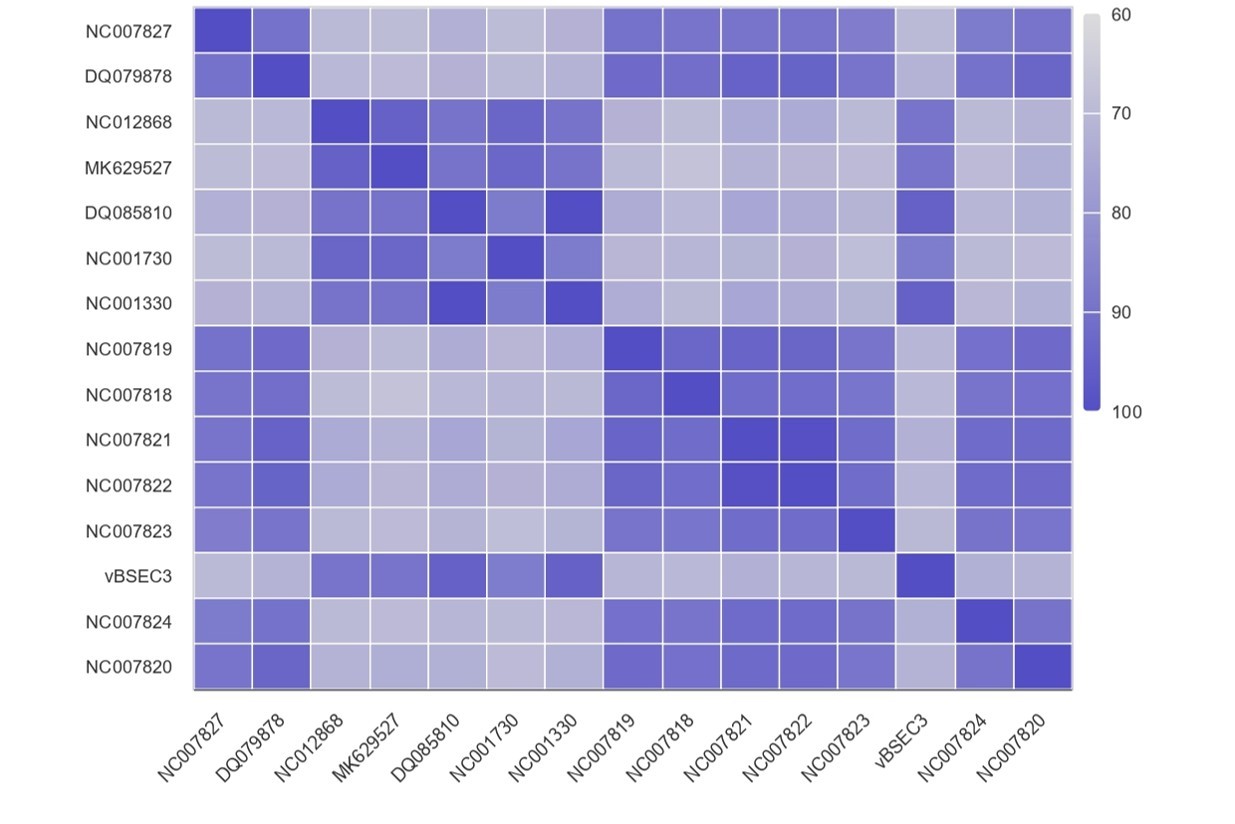

Supplement: Supplementary file 1 [file pathogens-15-00330-s001.zip › Supplementary Figure S1_600.jpeg]
